# *In situ* heating TEM observations on carbide formation and α-Fe recrystallization in twinned martensite

**DOI:** 10.1038/s41598-018-32896-z

**Published:** 2018-09-27

**Authors:** X. Liu, T. H. Man, J. Yin, X. Lu, S. Q. Guo, T. Ohmura, D. H. Ping

**Affiliations:** 1 School of Materials Science and Engineering, Dalian Jiaotong University, Dalian, 116028 China; 2 National Institute for Materials Science, Sengen 1-2-1, Tsukuba, 305-0047 Japan; 3 Department of Materials Physics and Chemistry, Kyushu University, 774 Motooka, Nishi-ku, Fukuoka 819-0395 Japan

## Abstract

The microstructural evolution of twinned martensite in water-quenched Fe–1.6 C (wt.%) alloys upon *in situ* heating was investigated using transmission electron microscopy (TEM). In the as-quenched samples, a high density of a body-centred cubic (bcc) {112} 〈111〉 -type twinning structure exists in the martensite structure. Upon *in situ* heating to approximately 200–250 °C, carbides (mainly θ-Fe_3_C cementite) accompanying a detwinning process were observed only in the originally twinned region. The carbides were absent in the originally untwinned (twin-free) region. The experimental results have suggested that the formation of the carbides depends on the twinning-boundary ω-Fe metastable phase, which can be stabilised by interstitial carbon atoms. When the specimens were heated, the twinning-boundary ω-Fe(C) transformed into carbide (mainly θ-Fe_3_C cementite) particles on the original {112} twinning planes. Further heating resulted in substantial recrystallisation of α-Fe fine particles, which formed immediately after martensite transformation. The results presented here should be helpful in understanding the microstructural evolution of various carbon steels.

## Introduction

Tempering is an important process in improving the properties of carbon steels. In particular, quenched high-carbon steel is usually brittle; a post-tempering or ageing process is necessary for improving the steel’s ductility for practical applications^1–8^. During tempering or ageing, the quenched martensite usually undergoes a highly complex microstructural evolution, including the formation of fine carbides, a detwinning process, dislocation-like substructure formation, recrystallisation^9,10^. A satisfactory understanding of the tempering behaviour has not yet been achieved^11^.

One of the main reasons for the aformentioned complexity is that the nature of the martensite substructure has not yet been fully understood^12,13^. Another reason is that the mechanism by which fine carbides form in martensite structure during tempering remains unclear.

Upon tempering of quenched high-carbon martensitic steels, the detwinning process and the formation of the carbides normally start at a relatively low-temperature: approximately 100 to 400 °C^7,14–17^. However, in low-carbon steels, the carbides (mainly cementite) normally form after tempering at approximately 400 °C^9,18^. In high-carbon steels, the quenched martensite or twinned martensite starts to decompose or transform at even lower temperature^19^. Such a transformation process involves carbide formation and the detwinning process, and the cementite carbides start to form preferably at the twinning boundary region^3^. The carbon atoms are generally assumed to be super-saturated in the martensite structure during quenching and then to diffuse to the twinning-boundary region to form cementite particles during post-tempering^3^. However, the reason why the twin boundaries are preferable sites for the carbide formation is still unclear.

Previous studies mainly focused on carbide formation during the tempering process, and most of the studied alloys were not simply Fe–C binary alloys^3^; and less attention has been paid to the morphology change of twinning structures during tempering. Some studies have revealed that the twinning structure vanishes and dislocations change into subgrain boundaries during tempering^7^. Recently, a new mechanism of the microstructural evolution has been proposed that the twinned martensite can be treated as freshly formed martensite immediately after martensitic transformation; and that the other structure can be regarded as the result of tempering (auto-tempering or post-tempering)^9,10^.

Recent systematic electron diffraction analysis has unambiguously revealed that a second metastable ω-Fe phase coexists with body-centred cubic (bcc) {112} 〈111〉 -type twin boundaries in a twinned martensite structure, which is a common substructure in high-carbon martensite^9,10,20–25^. The morphology of the ω-Fe phase, which exists only at the twin boundaries because this phase and the twin boundary structure stabilise each other, is fine-particle-like; and its particle size is only 1–3 nm^26^. Carbon atoms have been suggested to occupy the octahedral interstitial sites in the ω-Fe phase and to stabilise the ω-Fe fine particles^20^. Given the aforementioned nature of the twinned martensite, the tempering behaviour of high-carbon martensite, which normally has a bcc {112} 〈111〉 -type twinned structure as its substructure, should be revisited.

In the present work, to elucidate the transformation product of such a twinned martensite during tempering, *in situ* heating experiments were carried out on water-quenched high-carbon Fe–1.6 C (wt.%) binary alloys during transmission electron microscopy (TEM) observations. We selected the twinned martensite for the present investigation simply because of the absence of carbide in the twinned region^20,27^.

## Materials and Experiment

One 2-kg Fe–C (C: 1.6 wt.%) ingot was prepared in a high-vacuum induction furnace under an Ar atmosphere. The ingot was solution treated at approximately 1200 °C for 2 h and then hot forged into a thick plate with a thickness of approximately 20 mm. Small plates or bulk samples with approximate dimensions of 10 mm × 10 mm × 0.5–10 mm were mechanically cut and austenitised at 1150 °C for 0.5 h under flowing Ar and then water-quenched. The specimens for TEM observation were prepared from the water-quenched samples by mechanical grinding/polishing and subsequent ion-milling at room temperature. The *in situ* microstructural observations were carried out on a JEM 2000FX transmission electron microscope operated at 200 kV. In general, the temperature value recorded in the *in situ* TEM observations is higher than the actual temperature in the observed regions because the observed regions are at the thin edge of near a hole in the TEM specimen, whereas the temperature was measured at the TEM specimen holder instead of at the observed regions.

Since the omega-Fe particle size is about 1–2 nm, it is not large enough for carrying out the nano-beam technique to characterize the crystal structure of the omega-Fe particle. In our investigated Fe-C binary alloys, the carbon element cannot be measured accurately by STEM/EELS, and the carbon concentration measurement is difficult since the omega-Fe particles are at the twinning boundaries, which show curved features and quite close to each other (twin thickness is also at nanoscale distance). High resolution TEM observations are difficult to distinguish the omega-Fe particle from the alpha-Fe lattice images since both phases are overlapping or coherent in most crystal directions. The ultra-fine omega-Fe particles are fully overlapped by the alpha-Fe lattice image. Thus, it is quite difficult to separate the omega-Fe from alpha-Fe. Fortunately those ultra-fine omega-Fe particles have the same crystal orientation to alpha-Fe phase. Thus, there is no much difference between conventional TEM and advanced TEM in charactering the omega-Fe structure. One of the methods to improve the investigation on the ultra-fine omega-Fe is probably the observation of dark-field high resolution TEM images.

## Results

### As-quenched microstructure

**Figure 1:**
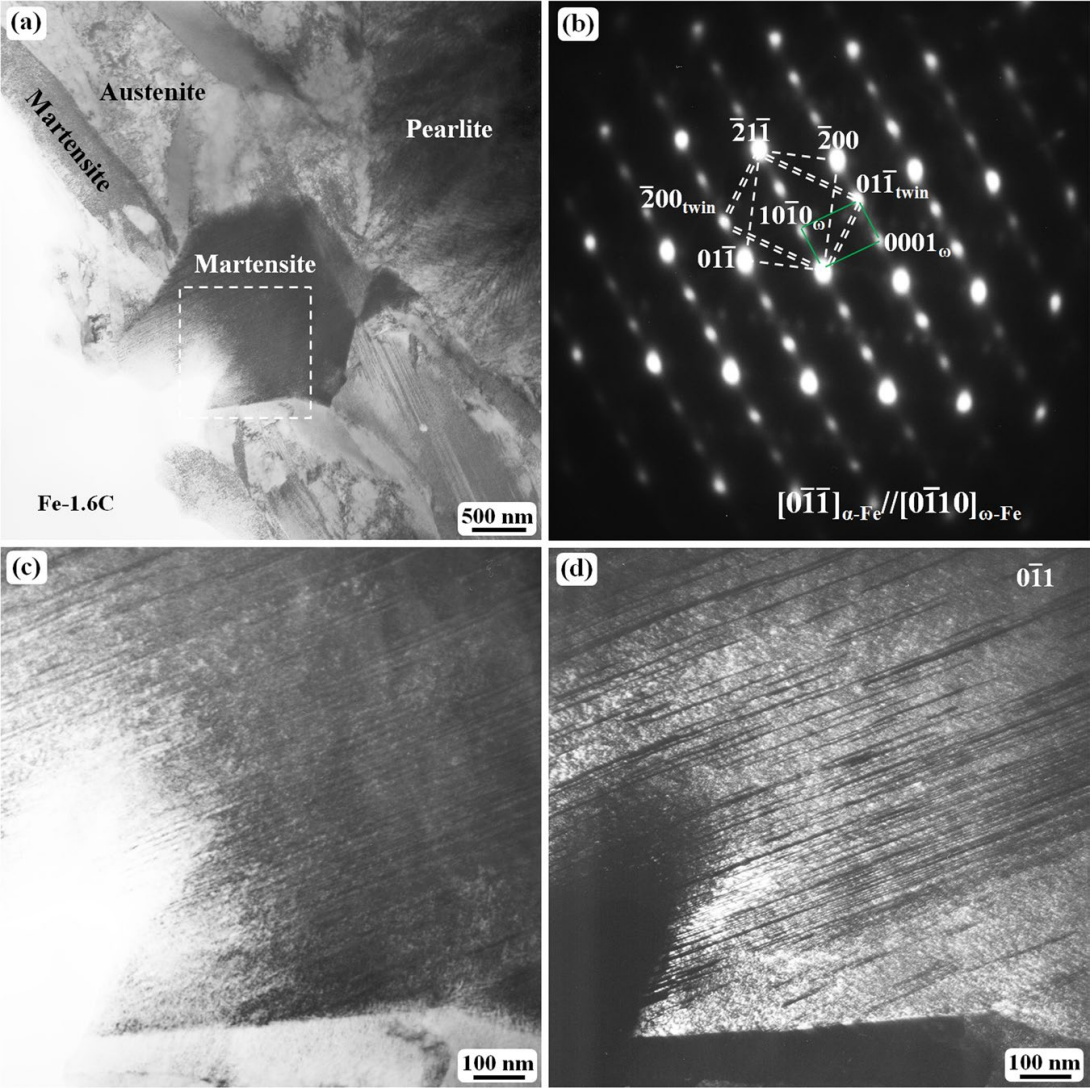
TEM images of the twinning structure in martensite plates: (**a**) bright-field image showing a general micrograph of Fe–1.6 C (wt.%) quenched at 1150 °C, (**b**) the selected area electron diffraction (SAED) pattern obtained from the internal region of the martensite indicated by dashed lines in (**a**,**c**) an enlarged image of the region outlined by dashed lines in (**a**,**d**) the corresponding dark field image of (**c**) obtained using the $01\bar{1}$ spot in (**b**).

Figure1 shows the general microstructure of the Fe–1.6 C (wt.%) samples water-quenched at 1150 °C. In the thin sample with a thickness of less than 2 mm, the microstructure mainly consists of martensite and austenite. As the sample thickness increases, pearlite structure becomes more and more obvious as revealed in Fig. 1a. Each martensite plate in the as-quenched state is composed of a high density of twinning substructure, which cannot be clearly observed at low magnification. The twinning structure can be confirmed to be bcc {112} 〈111〉 -type twin on the basis of the analysis of the selected area electron diffraction (SAED) pattern (Fig. 1b). Dark-field TEM observations clearly reveal the twin contrast (Fig. 1d), which is not easily observed in bright-field image (Fig. 1c) because of the high density of thin twins.

In the dark-field image (Fig. 1d), one can see that most of the twins are several nanometers thick, which normally causes an obvious diffraction streaking line along the $\bar{2}1\bar{1}$ direction as can be seen in Fig. 1b. Of course, the twin and the matrix crystals can cause double diffraction spots at the 1/3($\bar{2}1\bar{1}$) and 2/3($\bar{2}1\bar{1}$) positions; however, these double diffraction spots are fully overlapped with a metastable ω phase diffraction spots. The detail explanation about the ω phase can be found in the literature^20–25^, and the ω fine particles are confirmed to be located at the twinning boundary region only. At the same quenching temperature, the volume fraction of pearlite structure decreases as the sample thickness decreases.

### Carbides formation during *in situ* heating at 20–250 °C

**Figure 2:**
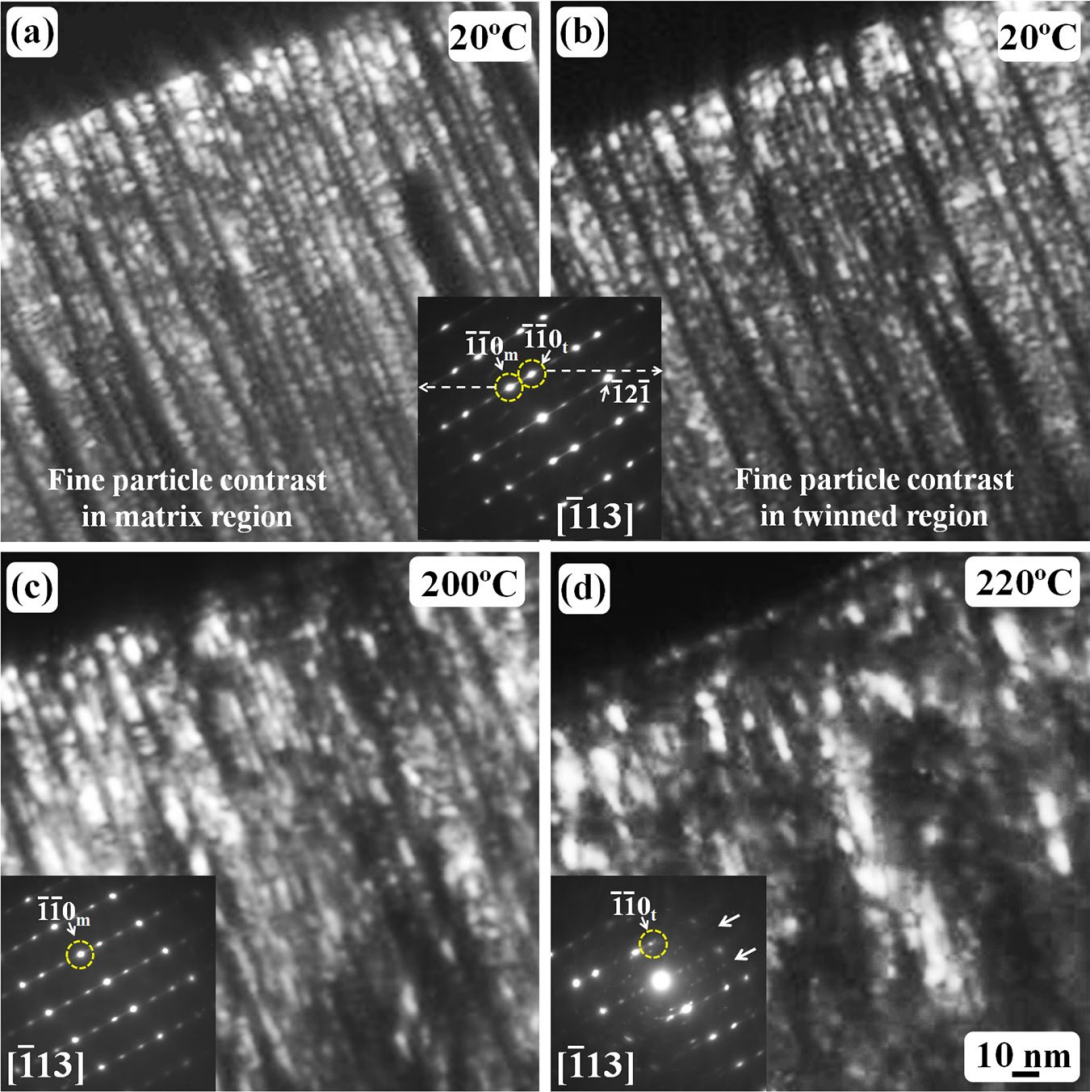
TEM dark-field images of the twinned structure in quenched Fe-1.6 C (wt.%) martensite: (**a**) dark-field image showing the fine structure of the matrix crystals; (**b**) dark-field image showing the fine structure of the corresponding twinned crystals in martensite, the SAED pattern of the twinned structure is inserted between (**a**–**c**) the dark-field image of the matrix crystals after the specimen was heated to 200 °C; (**d**) the dark-field image of the twinned crystals after the specimen was heated to 220 °C. The subscripts ‘m’ and ‘t’ denote matrix crystal and twin crystal, respectively. All figures have the same scale bar as that shown in (**d**).

Figure 2 shows a high-magnification of dark-field TEM observations on the twinned structure in martensite during an *in-situ* heating from room temperature to 220 °C. The observation area in Fig. 2 was the same region as shown in Fig. 1c, however, the specimen was tilted to one 〈113〉 direction. Figure 2a,b are the dark-field images obtained by using the matrix $\overline{11}{0}_{{\rm{m}}}$ and twin $\overline{11}{0}_{{\rm{t}}}$ diffraction spots, respectively. It is very clear that the thickness of matrix crystals and/or twin crystals is quite small, approximately 3 nm, and one more interesting point is that both matrix crystals and twin crystals have ultra-fine particle-like contrast, and the particle size is also approximately 2 or 3 nm. Since the electron diffraction patterns have revealed that the twinned martensite structure has a bcc {112} 〈111〉 -type twin, the ultrafine particles should correspond to fine α-Fe particles.

Such a fine-particle structure is stable from room temperature to 200 °C as revealed in the dark-field image and the corresponding SAED pattern (Fig. 2c). However, at 220 °C, the carbide diffraction spots could be seen as indicated by the white arrows in the corresponding SAED pattern in Fig. 2d, and the specimen started to tilt automatically due to the shrinking behavior of thin region in the TEM specimen. However, at this temperature, the twinned structure was still observed and the size of the dark-field particle-like contrast slightly increased, which suggests that the recrystallisation of the α-Fe particles started. Further heating resulted in a significant recrystallization of the ultra-fine α-Fe particles.

**Figure 3:**
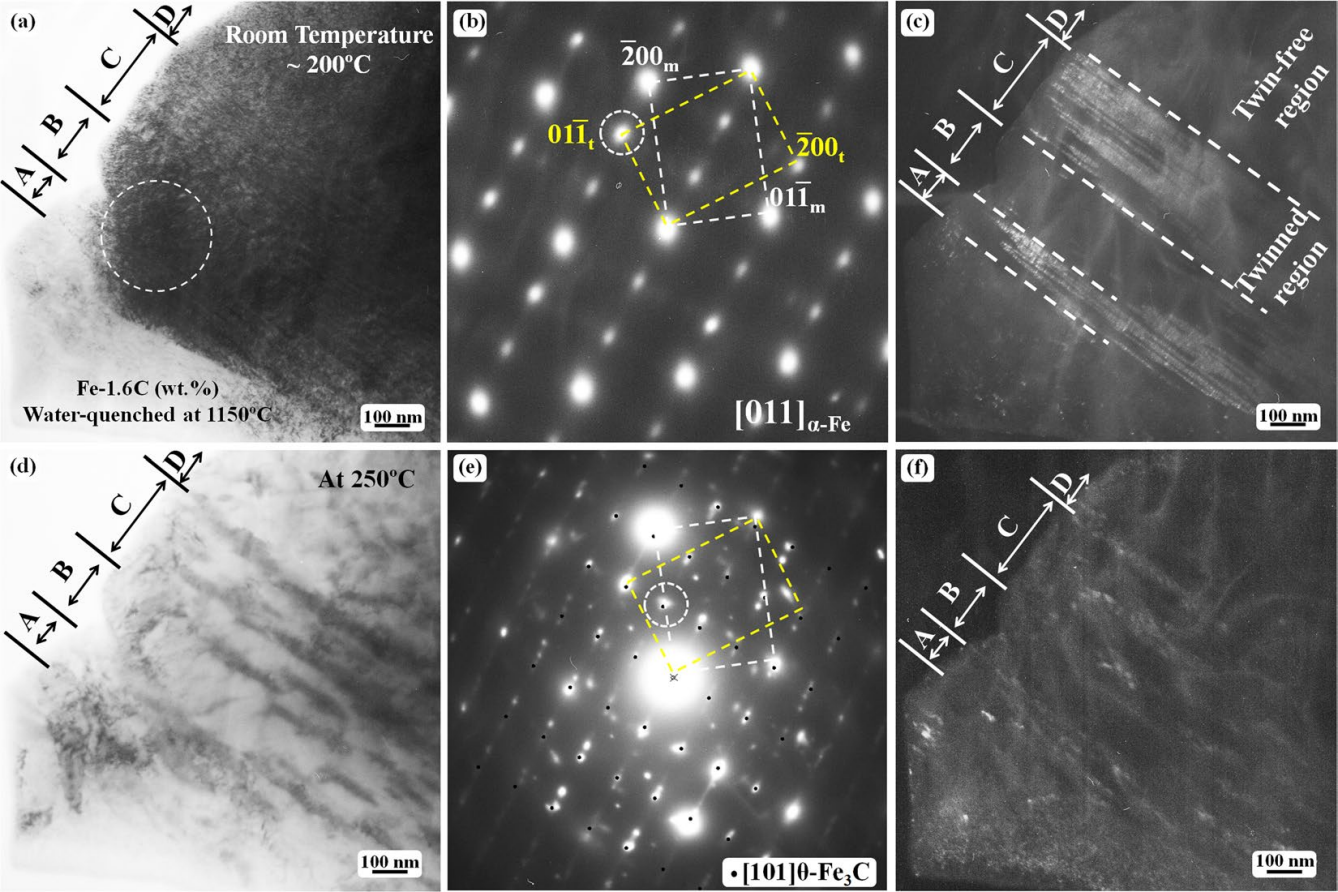
TEM observation of the twinned structure in martensite plates after *in situ* heating: (**a**) bright-field image showing a twinned structure; (**b**) the SAED pattern obtained from the outlined region indicated by a dashed circle in (**a**,**c**) the corresponding dark-field image taken using the $01{\bar{1}}_{{\rm{t}}}$ spot in (**b**,**d**) bright-field image of the same region as in (a) after the specimen was heated to 250 °C; (**e**) the SAED pattern from (**d**,**f**) the corresponding dark-field image of (**d**) obtained using the diffraction spot outlined by a dashed circle in (**e**). The calculated [101] zone axis diffraction spots of θ-Fe_3_C are superimposed on the experimental diffraction pattern in (**e**). The bright dots in (**f**) are carbide particles. The subscripts ‘m’ and ‘t’ denote matrix crystal and twin crystal, respectively.

*In situ* heating experiments were carried out on several TEM specimens because the experimental results could only be recorded for one local area during each heating process. Figure 3 shows TEM observation results of the microstructural evolution in the twinned martensite during an *in situ* heating of another TEM specimen. At this time, the carbide formation was carefully observed at above 200 °C. The twinning structure morphology did not obviously differ from room temperature to 200 °C during *in situ* heating TEM observations as shown in Fig. 3(a–c).

Figure 3(a) shows a bright-field TEM image of a partial twinned martensite heated up to 200 °C from the quenched state (room temperature). Figure 3(b) is the corresponding SAED pattern from the outlined region by the dashed circle in Fig. 3(a). The SAED pattern clearly reveals a bcc {112} 〈111〉 -type twin structure. Figure 3(c) is the dark field image taken by using the $01{\bar{1}}_{{\rm{t}}}\,$ diffraction spot in Fig. 3(b), thus, the bright contrast region corresponds to the twinned structure, and the dark contrast region is the twin-free area. In Fig. 3(a,c), the areas indicated by ‘A’ and ‘C’ are twinned, whereas the areas indicated by ‘B’ and ‘D’ are un-twinned or twin-free regions.

No carbides were observed in the quenched twinned martensite region. As previously explained, the extra diffraction spots at the 1/3($\bar{2}1\bar{1}$) and 2/3($\bar{2}1\bar{1}$) positions in Fig. 3b originate from the ω-phase particles distributed at the twinning-boundary region. When the sample was heated to 250 °C, carbide particles, which were identified as θ-Fe_3_C cementite, formed in the originally twinned region. The dark contrast in Fig. 3d and the bright contrast in Fig. 3f correspond to the cementite particles. In the SAED pattern (Fig. 3e), the diffraction spots from the [101] zone axis of θ-Fe_3_C (*a* = 4.524 Å, *b* = 5.088 Å, *c* = 6.741 Å^28^) are indicated by small black dots, which overlap some of the diffraction spots. Both the bright-field (Fig. 3d) and the dark-field (Fig. 3f) images clearly reveal that the carbides are absent in the twin-free regions. Interestingly, these carbides appear to be aligned with the ($\bar{2}1\bar{1}$) twinning plane. The contrast size of the carbide particles shown in the bright-field image (Fig. 3d) and dark-field image (Fig. 3f) differs. The carbide particles appear larger in the bright-field image, whereas they are small in the corresponding dark-field image. This result reveals that the larger carbides in the bright-field image are actually composed of numerous fine carbide particles with approximately the same orientation.

### Coarsening of carbides during *in situ* heating at 250–500 °C

**Figure 4:**
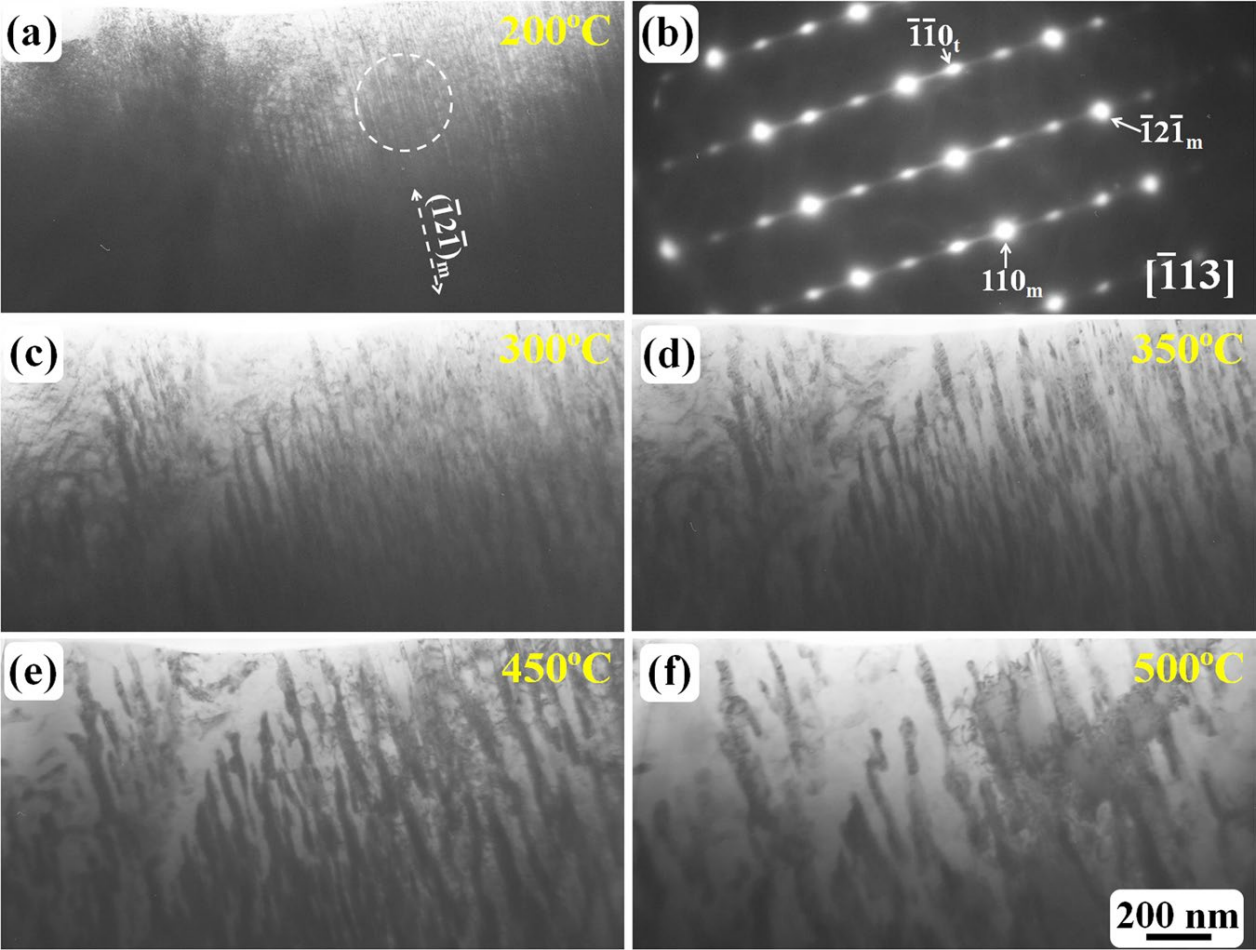
TEM observation of the coarsening of carbides during *in situ* heating: (**a**) carbides were not formed at 200 °C; (**b**) the SAED pattern obtained from the outlined region indicated by a dashed circle in (**a**,**c**–**f**) the morphology of carbides (θ-Fe_3_C particles) from 300–500 °C. All bright-field TEM images have the same scale bar as that shown in (**f**).

As revealed in Figs 2 and 3, carbides started to form at approximately 220 °C, the particle morphology of the carbides became obvious. However, the ω-Fe diffraction spots and the {112} 〈111〉 -type twin relationship were still observed. Further heating resulted in coarsening of the carbides. Figure 4 shows the heating results observed from different region in the same TEM specimen. At 200 °C, the twinning structure (Fig. 4a) and the ω-Fe phase (Fig. 4b) remained unchanged. At temperatures as high as 300 °C (Fig. 4c), fine carbide particles with a rod-like morphology (as confirmed by electron diffraction analysis in Fig. 3, these carbides are θ-Fe_3_C cementite particles) were aligned with the $\bar{1}2\bar{1}\,$ original twinning plane; these rod-like particles were gradually coarsened and tended to become spherical as revealed in Fig. 4f.

During the present *in situ* heating experiments, electron diffraction observation was carried out on the observed region at temperature intervals of 20 °C after the temperature was increased to 180 °C. The θ-Fe_3_C cementite was the only carbide clearly observed during the present investigation of high-carbon binary alloys.

### Recrystallisation of α-Fe nano-crystallites

**Figure 5:**
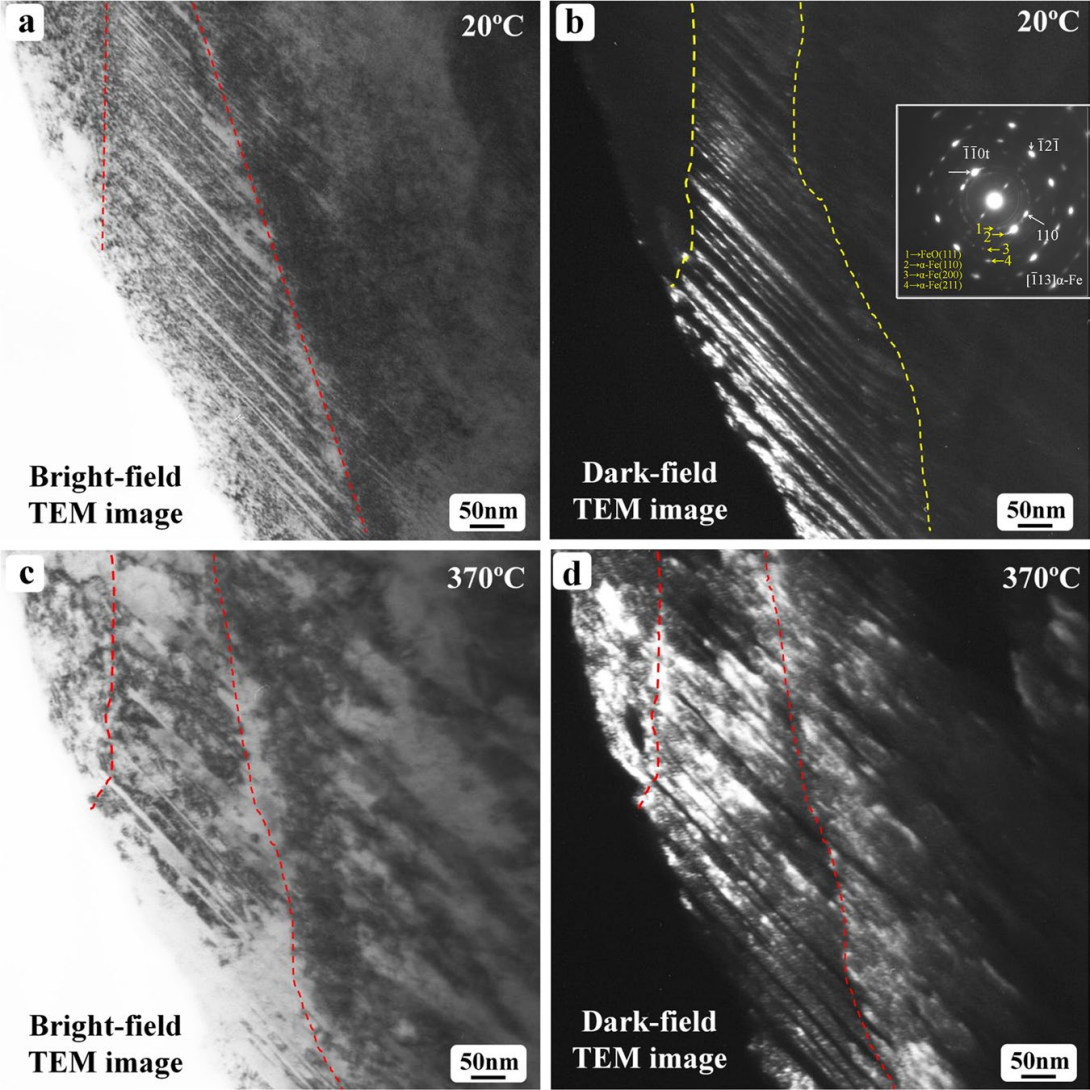
TEM observation of the as-quenched Fe-1.6 C sample after *in situ* heated to 370 °C: (**a**) bright-field and (**b**) dark-field images showing the twinned structure in as-quenched martensite; (**c**) bright-field and (**d**) dark-field images revealing the coarsening of the twinned crystals after *in situ* heated to 370 °C.

The above *in-situ* experimental observations focused on the carbide formation and coarsening process. The microstructural evolution of the matrix phase in martensitic structure during heating is shown in Fig. 5. Figure 5 shows TEM observation results of the *in-situ* heated specimen from room temperature to 370 °C. The thickness of the twinned crystals was less than 10 nm in the observed region (Fig. 5a,b) of the as-quenched state. At 370 °C, the thickness of the twinned crystals was significantly larger than that in the as-quenched state (Fig. 5c,d). As revealed in Fig. 2, the matrix or twinned crystal is composed of ultra-fine α-Fe particles with almost the same orientation in as-quenched state, thus, the coarsening of these matrix or twinned crystals is reasonably treated as a recrystallization process to reduce the number density of grain boundaries or sub-grain boundaries formed by those ultra-fine particles. Interestingly, the coarsening appears perpendicular to the twin boundary plane. During heating, the twin boundary planes may also move through a transition of bcc ↔ ω lattice^25^.

Several diffraction rings can be seen clearly in the inserted diffraction in Fig. 5b. Some of these rings are indicated by Arabic numerals 1–4. The smallest ring, which is actually artifact caused by the TEM selected aperature, is also visible. The number 1 ring can be indexed as the (111) diffraction of FeO, which has a face-centered-cubic structure with a lattice parameter of 4.292 Å. The 2, 3 and 4 rings can be indexed as the (110), (200) and (211) diffraction of α-Fe, respectively. During the TEM specimen preparation, ion-milling was applied. Thus, the fine particle re-deposition onto the specimen surface happened during ion-milling. Those fine particles (α-Fe) were from the TEM specimen itself. It is natural for some of the α-Fe fine particles to get oxidized and to form FeO particles. The existence of these diffraction rings does not affect our investigation results on the ω → θ carbide transition.

**Figure 6:**
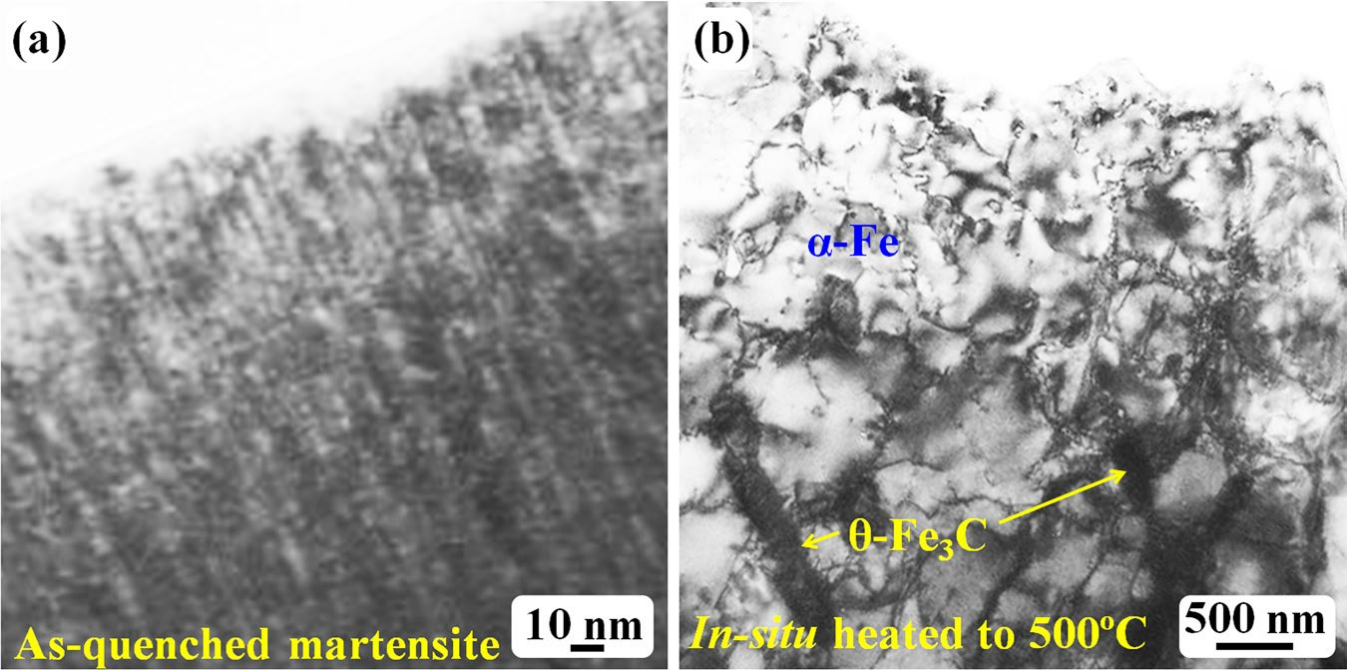
TEM bright-field images: (**a**) as-quenched martensite; (**b**) *in-situ* heated to 500 °C.

After the *in situ* heating temperature reached 500 °C, the TEM specimen was then cooled to room temperature and installed into a double-tilting TEM holder for tilting experimental observations to confirm the carbide structure and morphology. Figure 6 shows TEM bright-field observation of the *in situ* heated specimen up to 500 °C. Figure 6a shows the bright-field image that corresponds to the dark-field images in Fig. 2a,b. This image shows the interior of the as-quenched martensite structure with twinning-structure contrast. In the bright-field mode, the contrast of ultrafine particles or dots is evident (Fig. 6a); and these fine particles are α-Fe crystallites. After the specimen was heated *in situ* to 500 °C, significantly coarsened grains with many subgrain boundaries were clearly observed (Fig. 6b). These coarsened grains were formed through a recrystallization process during the *in situ* heating.

**Figure 7:**
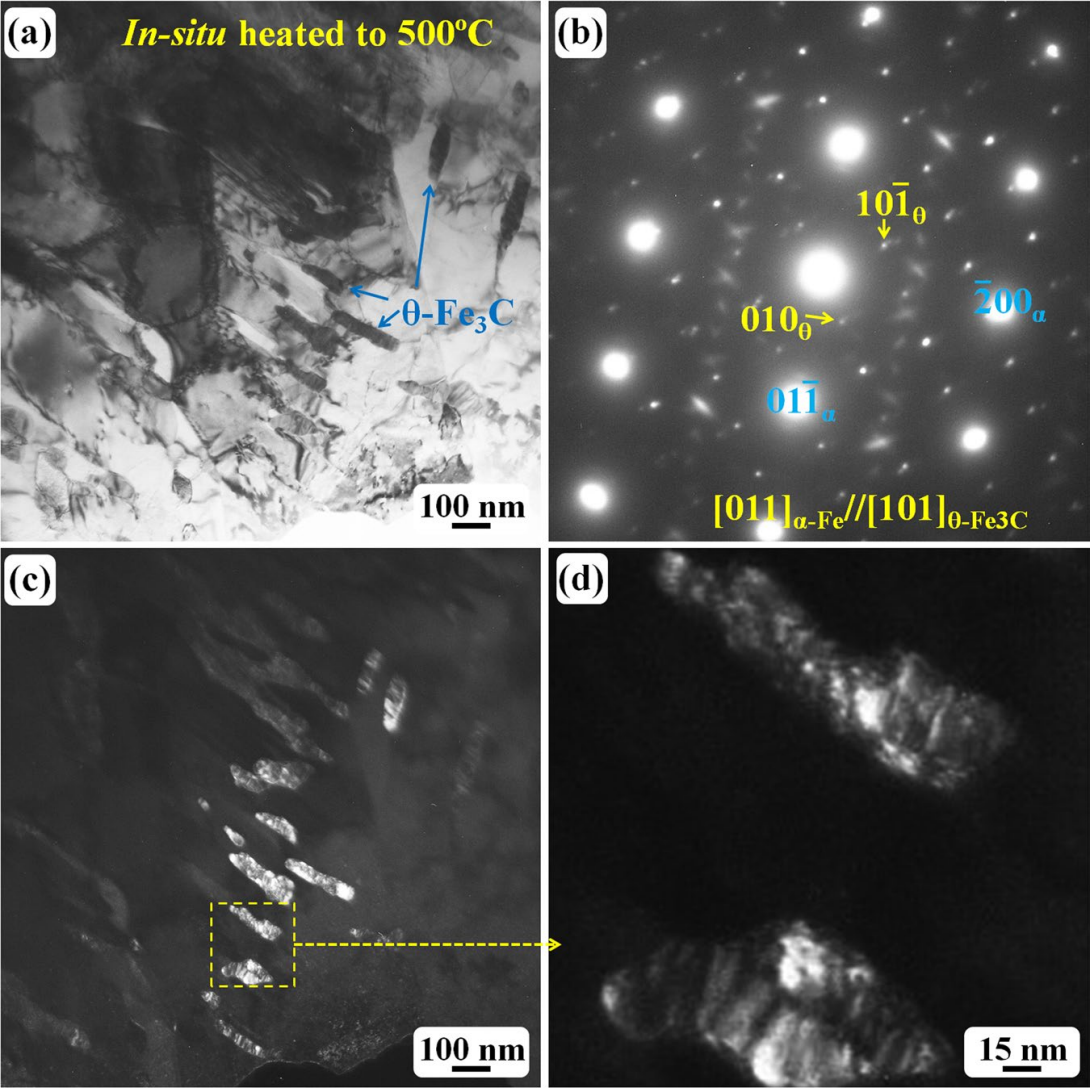
TEM observation of the specimen after *in situ* heated to 500 °C: (**a**) bright field image showing large carbides; (**b**) the corresponding SAED pattern revealing the carbide is θ-Fe_3_C; (**c**) the dark field image showing the carbide morphology; (**d**) enlarged dark field image revealing the larger carbide contains a lot of fine particles.

Figure 7a reveals another region with carbides and α-Fe grains after the recrystallisation. The corresponding SAED pattern (Fig. 7b) has confirmed that the matrix grains are α-Fe and the black rod-like particles are carbides. The diffraction patterns in Figs 7b and 3e are quite similar. Electron diffraction analysis has confirmed that the carbides in Fig. 7a are θ-Fe_3_C cementite particles with the same lattice parameters as shown in Fig. 3e. Once the θ-Fe_3_C cementite formed, the temperature increase did not change the carbide structure, just the morphology. As shown in Fig. 7c, the carbides became much larger than that in lower temperature, and the larger carbides are well-separated, and the rod-like morphology is still dominant. Two large carbides in Fig. 7c have been enlarged and shown in Fig. 7d. These carbides seem to be composed of a lot of ultrafine particles. In Fig. 7b, two sets of diffraction spots have been indexed as α-Fe and θ-Fe_3_C phases. However, some weak diffraction spots are still visible. Those weak spots belong to a new carbide (hcp structure, *a* = *b* = 0.573 nm, *c* = 1.206 nm) and have been characterized by Tirumalasetty *et al*. recently^29^.

## Discussion

The formation mechanism of carbides in martensite in carbon steels has been commonly explained on the basis of assumed nucleation and carbon diffusion^3^. The carbon atoms have been suggested to be solid-solutioned in α-Fe crystals and to remain at the octahedral interstitial site of the bcc structure by assuming a body-centred tetragonal (bct) crystalline structure of martensite^30–36^. During the present *in situ* TEM heating experiments, the thickness of the TEM specimens at the observation region is normally on the order of several nanometres to tens of nanometres. In general, TEM specimen chambers are under a high vacuum of approximatelt 10^−5^ Pa. The carbon atoms were indeed inside the whole martensite structure because of the late-formed carbides. The formation of fine carbide particles during heating strongly suggests that the carbon atoms do not freely diffuse in the solid specimen or in the martensite structure^25^.

In fact, the twinned structure is the substructure of high-carbon martensite; thus, twin boundaries are abundant in each martensite structure. Twinning boundaries in martensite have been previously regarded as potent nucleation sites for cementite formation^3,27^. This assumption leads to a simple question: Why do the carbon atoms preferentially diffuse to the twinning boundaries instead of normal grain boundaries or the specimen free surface? Is an attractive force associated with the twinning boundary? If not, then one possibility is that the carbon atoms are originally at the twinning-boundary region together with the ω-Fe particles.

In simple terms, carbon atoms can remain in the ω-Fe particles at the twinning boundaries, which have been confirmed by recent theoretical calculation results^20,26^. These ω-Fe particles with carbon atoms transform into cementite particles during tempering, and the twinning boundaries then change to normal grain boundaries or subgrain boundaries through detwinning and α-Fe recrystallisation processes^9,10,37^. Thus, the experimental results revealed in Fig. 3 can be reasonably explained. The carbide particles are formed only in the twinned region at twinning boundaries (the ω-Fe particles) during heating, whereas the carbides are absent in the twin-free region because no carbon atoms and no twin boundaries or ω-Fe particles are located there.

Previous analyses based on the crystallographic transition and theoretical calculations have suggested that the ω-Fe structure can form in the face-centred cubic (fcc) γ-Fe phase^12,38,39^ and that the transformation of γ-Fe to α-Fe can occur through the following process: γ-Fe(C) → ω-Fe(C) + α-Fe. The ω-Fe(C) has been suggested and observed to be distributed at the twin boundary region as fine particles^10,25^. During the *in situ* heating, ω-Fe(C) can transform into θ-Fe_3_C cementite naturally because the energy barrier of the ω-Fe(C) → θ-Fe_3_C transformation is quite small^20^. Because a unit cell of the hexagonal ω-Fe phase has three iron atoms, the ω-Fe(C) unit cell naturally has the Fe_3_C chemical composition after one carbon atom joins in. This unit cell arrangement is also one of the reasons why θ-Fe_3_C cementite particles can form at relatively low temperatures.

The relationship among carbon atoms, the ω-Fe structure, and the twin boundaries (bcc {112} 〈111〉 -type twin) can be summarised as follows: Carbon atoms stabilise the ω-Fe structure; and the ω-Fe structure and the twin boundary stabilise each other. After the θ-Fe_3_C cementite forms, the original ω-Fe(C) naturally disappears; the twinning structure then also disappear through the recrystallisation process^10^ of the fine α-Fe particles, as shown in Fig. 2. During the recrystallisation process or subgrain boundary migration, several fine θ-Fe_3_C cementite particles may connect each other and merge into a single larger particle^10^. This recrystallisation mechanism is reasonable because the solid–solid transitions of γ-Fe(C) → ω-Fe(C) + α-Fe and ω-Fe(C) → θ-Fe_3_C result in the surrounding α-Fe or θ-Fe_3_C fine particles having approximately the same orientation in a local region.

## Conclusion

TEM *in-situ* heating observations of water-quenched high-carbon martensite have revealed the following facts:

1. The carbides formed in twinned regions and the carbide particles were aligned with the original {112} twinning planes. Because the ω-Fe fine particles were at the twinning boundaries in the as-quenched state and because its diffraction spots disappeared upon heating, the ω-Fe particles are reasonably suggested to be transformed into carbides upon tempering. The carbide particles transformed from the ω-Fe phase are the θ-Fe_3_C cementite. The ω-Fe phase particles at twinning-boundary regions can be considered a precursor to the θ-Fe_3_C cementite during martensite tempering.
2. The θ-Fe_3_C cementite is the only carbide transformed from the ω-Fe(C) in high carbon martensite, and the structure remains unchanged until 500 °C.
3. The coarsening of the carbide particles can occur through the aggregation of fine carbide particles during the recrystallisation process of α-Fe grains.
